# Association between the *ERCC5* Asp1104His Polymorphism and Cancer Risk: A Meta-Analysis

**DOI:** 10.1371/journal.pone.0036293

**Published:** 2012-07-18

**Authors:** Mei-Ling Zhu, Mengyun Wang, Zhi-Gang Cao, Jing He, Ting-Yan Shi, Kai-Qin Xia, Li-Xin Qiu, Qing-Yi Wei

**Affiliations:** 1 Department of Oncology, Fudan University Shanghai Cancer Center, Shanghai, China; 2 Department of Oncology, Shanghai Medical College, Fudan University, Shanghai, China; 3 Cancer Research Laboratory, Fudan University Shanghai Cancer Center, Shanghai, China; 4 Department of Breast Surgery, Cancer Center and Cancer Institute, Fudan University, Shanghai, China; 5 Department of Epidemiology, The University of Texas MD Anderson Cancer Center, Houston, Texas, United States of America; IPO, Inst Port Oncology, Portugal

## Abstract

**Background:**

Excision repair cross complementing group 5 (*ERCC5* or *XPG*) plays an important role in regulating DNA excision repair, removal of bulky lesions caused by environmental chemicals or UV light. Mutations in this gene cause a rare autosomal recessive syndrome, and its functional single nucleotide polymorphisms (SNPs) may alter DNA repair capacity phenotype and cancer risk. However, a series of epidemiological studies on the association between the *ERCC5* Asp1104His polymorphism (rs17655, G>C) and cancer susceptibility generated conflicting results.

**Methodology/Principal Findings:**

To derive a more precise estimation of the association between the *ERCC5* Asp1104His polymorphism and overall cancer risk, we performed a meta-analysis of 44 published case-control studies, in which a total of 23,490 cases and 27,168 controls were included. To provide additional biological plausibility, we also assessed the genotype-gene expression correlation from the HapMap phase II release 23 data with 270 individuals from 4 ethnic populations. When all studies were pooled, we found no statistical evidence for a significantly increased cancer risk in the recessive genetic models (His/His vs. Asp/Asp: OR = 0.99, 95% CI: 0.92–1.06, *P* = 0.242 for heterogeneity or His/His vs. Asp/His + Asp/Asp: OR = 0.98, 95% CI: 0.93–1.03, *P* = 0.260 for heterogeneity), nor in further stratified analyses by cancer type, ethnicity, source of controls and sample size. In the genotype-phenotype correlation analysis from 270 individuals, we consistently found no significant correlation of the Asp1104His polymorphism with *ERCC5* mRNA expression.

**Conclusions/Significance:**

This meta-analysis suggests that it is unlikely that the *ERCC5* Asp1104His polymorphism may contribute to individual susceptibility to cancer risk.

## Introduction

Exposure to environmental carcinogens can cause different types of DNA damage that subsequently lead to carcinogenesis of different tissues, if left unrepaired. During the evolution, humans have developed a versatile DNA repair machinery to ensure genome integrity in response to the insults of cancer-causing agents. DNA repair is a complex biological process consisting of several distinct pathways. To date, more than 150 human DNA repair genes have been identified in five major pathways: nucleotide excision repair (NER), base excision repair (BER), mismatch repair (MMR), double-strand break repair (DSBR), and transcription coupled repair (TCR). Of those pathways, NER is the most studied DNA repair mechanism responsible for various types of DNA damage, including thymidine dimers, oxidative DNA damage, bulky adducts cross-links, and alkylating damage [1]. At least eight core genes (i.e., *ERCC1, XPA, XPB/ERCC3, XPC, XPD/ERCC2, XPE/DDB1, XPF/ERCC4,* and *XPG/ERCC5*) in the NER pathway play vital roles in repairing DNA damage and maintain genome integrity [2], [3].

**Figure 1:**
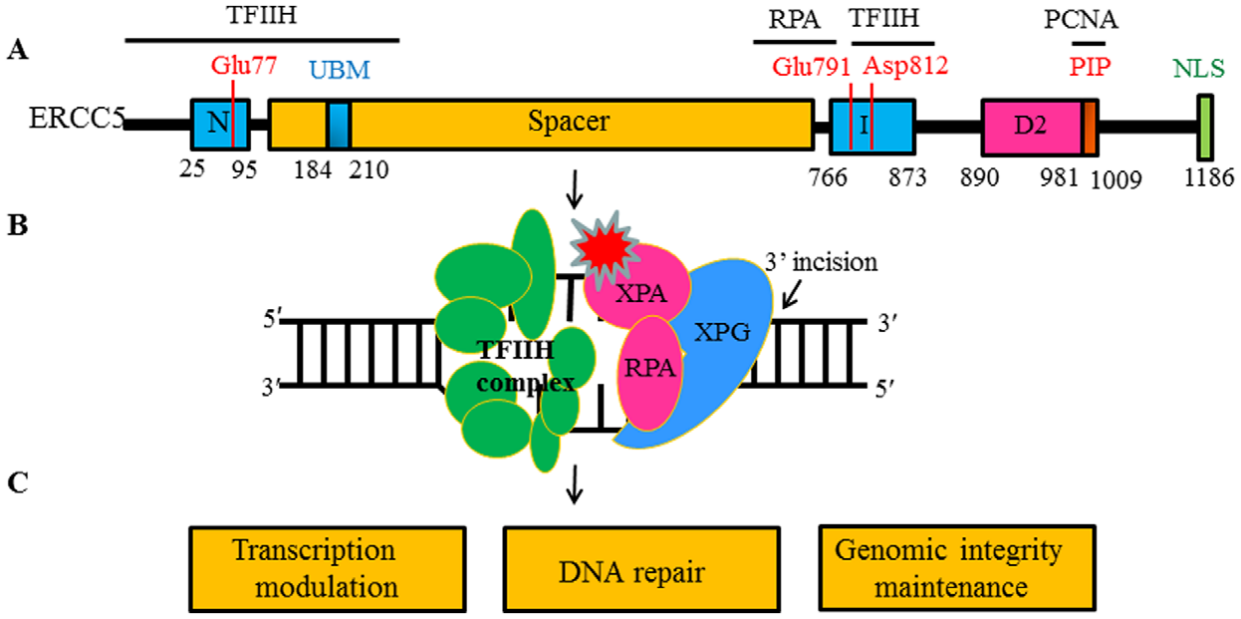
Structural characteristics and function of *ERCC5* protein modified from the picture in the reference [14]. (A) Human ERCC5 protein harbors the N- and I-nuclease domains (blue) and 600 amino acid spacer region (Orange). Conserved residues (Glu77, Glu791 and Asp812) located in the active site (red). Interaction regions with TFIIH, RPA, and PCNA (PIP) and the ubiquitin-binding domain (UBM) are indicated. (B) ERCC5 cleaves 5′ flap, splayed-arm and bubble substrates at ss/dsDNA junctions and makes the 3′ incision in NER. (C) ERCC5 protein plays versatile roles in cellular processes including DNA repair, genomic integrity maintenance and modulation of gene transcription.

The excision repair cross complementing group 5 (*ERCC5*) gene, also known as the xeroderma pigmentosum group G (*XPG*) gene, is a member of the flap structure-specific endonuclease 1 (FEN1) family and encodes a protein of 1186 amino acids. The primary structure of human *ERCC5* protein harbors the N- and I-nuclease domains that are highly conserved, which together form the nuclease core [4]. Mutations of several conserved residues in the active site, including Glu77, Glu791 and Asp812, abolish the catalytic activity of the protein [5], [6]. N- and I-nuclease domains are separated by the 600 amino acid spacer region that is highly acidic for protein-protein interactions including with TFIIH [7], [8], [9] and RPA [10] and therefore recruits ERCC5 to the sites of NER [11]. Additionally, ERCC5 contains ubiquitin-binding motif (UBM) as well as a PIP domain that mediates interactions with PCNA [12], [13]. Such an interaction between ERCC5 and PCNA could be involved in triggering the 3′ incision in NER. ERCC5 cleaves the 5′ flap, splayed arm and a variety of bubble substrates at ss/dsDNA junctions with the 5′overhang and makes the 3′ incision in NER [14] (**Figure 1**).

As a structure-specific endonuclease and also a 5′-3′ exonuclease, the ERCC5 protein is required for two sub-pathways in NER. One is TCR, which preferentially removes DNA damage in the transcribed DNA strand of active genes; the other is global genomic repair (GGR), which removes lesions throughout the genome [15], [16]. Additionally, ERCC5 also possesses some secondary functions independent of its cleavage activity in supporting a role of TFIIH in receptor-mediated transcription [17], [18]. Furthermore, data from S. cerevisiae studies demonstrate a role for Rad2 (the ERCC5 homolog) in RNA polymerase II-mediated transcription [19]. In addition, ERCC5 is thought to have a possible role in the removal of oxidative damage by BER and possibly other pathways [20], [21]. Numerous studies using various tumor cell lines or tissues indicates that *ERCC5* plays a key role in carcinogenesis and that its deficiency leads to DNA repair defects, genomic instability, failure of modulation of gene transcription [22]–[26]. Genetic disorders resulting from mutations in the *ERCC5* gene, such as xeroderma pigmentosum (XP), Cockayne syndrome (CS), and tri-chothiodystrophy (TTD), underscore the biological importance of this gene [14], and most of these syndromes follow a recessive genetic model, in which heterozygotes are unaffected, but mutant homozygotes manifest the disease [27].

The *ERCC5* gene is located on chromosome 13q22-q33, consists of 15 exons that range from 61 to 1074 bp and 14 introns that range from 250 to 5763 bp, and spans 32 kb [28]. To date, at least 73 non-synonymous SNPs (nsSNPs) in the *ERCC5* coding region have been identified (http://www.ncbi.nlm.nih.gov/SNP/), and 24 SNPs in the gene region have been studied for their association with cancer risk (**Table S1**), of which only four were nsSNPs (**Figure S1**). For example, the Asp1104His polymorphism (rs17655 G>C) is common [minor allele frequency (MAF) >0.05] and regarded as a tagger, which was most frequently investigated for its association with cancer risk. Interestingly, relatively few nsSNPs are present in the eight NER core genes, suggesting the conservativeness of these genes for their biological importance.

In a published meta-analysis, a total of 12 SNPs of the eight NER core genes, including 6 nsSNPs, have been investigated for the associations with cancer risks [29]–[44], among which 5 nsSNPs were found to be associated either with risk of a specific cancer or risk of the overall cancer risks mostly under recessive genetic models (**Table S2**), but no published meta-analysis has summarized all reported studies of the Asp1104His polymorphism in association with risk of all cancer types. It is biologically plausible that the Asp1104His polymorphism, causing a change from aspartate to histidine at codon 1104 in *ERCC5* protein, may result in an alteration of the gene function, thus likely altering risk of developing cancers, possibly following a recessive genetic model.

To date, although a number of studies have been performed to investigate the association between the *ERCC5* Asp1104His polymorphism and cancer risk, the evidence regarding the role of SNPs of the *ERCC5* gene as a genetic marker for cancer risk is conflicting, partially because of the possible lack of a main effect of the SNP on risk of any single type **o**f cancer, a possibly low penetrance or weak effect, or the relatively small sample size in each of published studies. Therefore, we performed a meta-analysis to identify statistical evidence for an association between the *ERCC5* Asp1104His polymorphism and cancer risk using all published data to date.

## Materials and Methods

### Identification and Eligibility of Relevant Studies

We searched two electronic databases (MEDLINE and EMBASE) for all relevant articles with the following terms: “*ERCC5*” or “*XPG*”, “DNA repair”, “polymorphism” or “variant”, “case-control”, “risk”, “association”, and “cancer” or “carcinoma” or “neoplasm” or “malignance” (last search was updated on Sept 1, 2011). References of the retrieved articles or reviews on this topic were also manually screened for additional relevant eligible studies.

We defined inclusion criteria as follows: written in English or Chinese; case-control design; sufficient information for estimating odds ratio (OR) and their 95% confidence interval (CI); observed genotype frequencies in the controls in agreement with Hardy-Weinberg equilibrium (HWE). Abstracts and unpublished reports were not considered. Investigations in subjects with family history or cancer-prone disposition were also excluded. Meanwhile, if studies had overlapping subjects, we selected the most recent study that included the largest number of individuals in the publications. Additionally, we also checked for minor allelic frequency (MAF) among studies by different genotype frequencies in ethnic groups based on Hapmap or dbSNP frequencies reported for the different ethnic groups, and the datasets were excluded if they had a very high probability of inaccurate reported.

### Data Extraction

Two investigators (Zhu ML and Wang MY) independently extracted the following information from each study: the first author, year of publication, country of origin, ethnicity, cancer type, source of controls (population-based, hospital-based, family-based and mixed controls), genotyping method, number of genotyped cases and controls, numbers of genotypes for *ERCC5* Asp1104His (rs17655) in cases and controls, and main findings. For studies including subjects of different ethnic groups, we extracted data separately for each ethnic group and categorized as Caucasian, Asian, African and others. When a study did not state what ethic groups were included or if it was impossible to separate participants according to the data presented, we termed the sample as ‘others’.

### Correlation Analysis of *ERCC5* mRNA Expression

We provide biological plausibility of the studied SNP, we downloaded the Asp1104His genotyping data from the HapMap phase II release 23 data set consisting of 3.96 million SNP genotypes from 270 individuals from four populations (CEU: 90 Utah residents from northern and western Europe; CHB: 45 unrelated Han Chinese in Beijing; JPT: 45 unrelated Japanese in Tokyo; YRI: 90 Yoruba in Ibadan, Nigeria) (http://www.sanger.ac.uk/humgen/hapmap3) [45]. The data on *ERCC5* mRNA expression levels from EBV-transformed B lymphoblastoid cell lines from the same 270 HapMap individuals were available online (http://app3.titan.uio.no/biotools/tool.php?app=snpexp) as well [46], [47]. Then, we conducted linear regression model-trend test for assessing the correlation between Asp1104His and *ERCC5* mRNA expression for different populations.

### Statistical Methods

We assessed the association between the *ERCC5* Asp1104His polymorphism and cancer risk by crude ORs and 95% CIs. Then, we calculated the pooled ORs and 95% CIs under an assumption of a recessive genetic model (His/His vs. Asp/Asp or His/His vs. Asp/His + Asp/Asp). In addition, we performed stratification analyses by cancer type (if one cancer type contained less than three studies, it was merged into the ‘other cancers’ group), ethnicity, source of controls, study design and sample size (<500, 500–1000, and >1000).

We evaluated the between-study heterogeneity by using the Chi square-based Q-test, which was considered significant if *P*<0.10. Values from single studies were combined using models of both random effects (DerSimonian and Laird method 1986) [48] and fixed effects (Mantel–Haenszel method) [49]. When *P* value of the heterogeneity test was >0.10, the fixed-effects model was used, which indicates no significant heterogeneity of the effect size across all studies; otherwise, the random-effects model was more appropriate, which tends to provide wider CIs, when the results of the constituent studies differ among themselves. To evaluate the effect of individual studies on overall risk of cancers, we conducted sensitivity analyses by excluding each study individually and recalculating the ORs and 95% CI. We also used the inverted funnel plot and the Egger’s test to examine the potential influence of publication bias (linear regression analysis) [50]. HWE among controls for each study was examined by the Pearson's goodness-of-fit chi-square test. The boxplot presentation and trend tests were performed with Statistical Analysis System software (v.9.1 SAS Institute, Cary, NC) All other statistical analyses were carried out with STATA software, version 11.0 (Stata Corporation, College Station, TX). All *P* values were two-sided with a significance level of 0.05, unless specified otherwise.

## Results

### Study Characteristics

We identified a total of 74 relevant publications after initial screening. Among these, 62 publications had met the inclusion criteria and were subjected to further examination. We excluded 8 publications because they did not present detailed genotyping information [51]–[58]. We also excluded 3 publications because they included the overlapped data with those included in the analysis [59], [60], [61]. Furthermore, we removed 7 publications because their genotype distributions among the controls deviated from HWE [62]–[68]. Therefore, our final data pooling consisted of 44 publications [69]–[112] with a total of 23490 cancer cases and 27168 controls, of which there were actual 49 case-control studies, because 5 publications provided more than one individual study **(Figure S2).** These 49 studies included 9 breast cancer studies, 8 skin cancer studies, 5 lung cancer studies, 5 bladder cancer studies, 3 head and neck cancer studies, 3 colorectal cancer studies, 3 non-Hodgkin lymphoma studies, and 13 studies of other cancers. Of these, there were 27 hospital-based studies, 20 population-based studies, 1 family-based study, and 1 study with mixed controls. In addition, 29 of 49 studies were conducted in Caucasians, 4 were conducted in African-Americans, 6 were conducted in Asians, and the remaining 10 were conducted in other ethnic groups. Several genotyping methods were used, including the polymerase chain reaction–restriction fragment length polymorphism (PCR-RFLP), which was the most frequently used method, TaqMan, sequencing, Illumina, SNaPshot, SNPlex and Mass spectrometry, but two publications did not provide information about genotyping methods. Additionally, all studies were in keep with HapMap or dbSNP frequencies reported for the different ethnic groups (**Table S3**).

### Quantitative Synthesis

When all eligible studies were pooled into one dataset for the meta-analysis, we found no statistical association between the *ERCC5* Asp1104His polymorphism and overall cancer risk under the recessive genetic models: His/His vs. Asp/Asp: OR = 0.99, 95% CI: 0.92–1.06 or His/His vs. Asp/His + Asp/Asp: OR = 0.98, 95% CI: 0.93–1.03.

**Figure 2:**
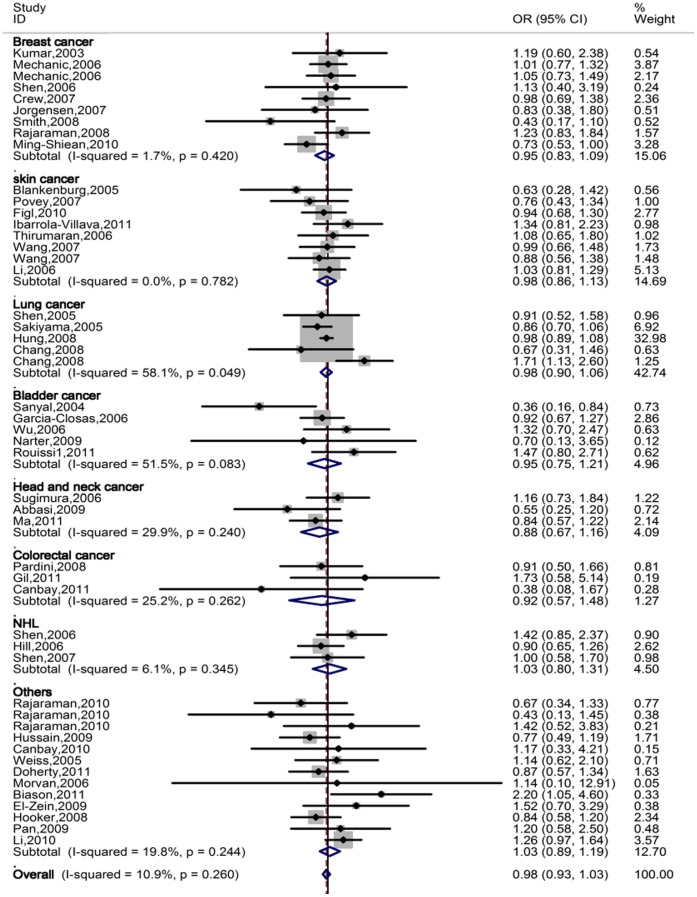
Forest plot of overall cancer risk of different cancer types associated with *ERCC5* Asp1104His polymorphism (His/His vs. Asp/His + Asp/Asp) by the fixed effects for each of the 44 published studies. For each study, the estimates of OR and its 95% CI were plotted with a box and a horizontal line. The symbol filled diamond indicates pooled OR and its 95% CI.

In the stratified analysis by ethnicity, we did not observe any association between the polymorphism and cancer risk in the recessive genetic models, neither and had the similar results in the stratified analyses by tumor type, source of controls, and sample size in cases (**Table 1** **,** **Figure 2**).

**Table 1:** Meta-analysis of the association between the *ERCC5* Asp1104His polymorphism and cancer risk under the XP recessive genetic model for 49 studies.

Variables	No. of studies	No. of subjects Cases/controls	His/His vs. Asp/Asp			His/His vs. Asp/His+Asp/Asp		
			OR (95%CI)^c^	*P*_OR_ a	*P*_het_ b	OR (95%CI)^c^	*P*_OR_ a	*P*_het_ b
**All**	49	23490/27168	0.99 (0.92–1.06)	0.707	0.242	0.98 (0.93–1.03)	0.393	0.260
**Cancer type**								
Breast	9	4915/5277	0.99 (0.85–1.15)	0.882	0.362	0.95 (0.83–1.09)	0.497	0.420
Skin	8	3909/4263	0.95 (0.78–1.16)	0.622	0.586	0.98 (0.86–1.13)	0.809	0.782
Lung	5	4702/5654	0.99 (0.86–1.15)	0.937	0.032	0.98 (0.90–1.06)	0.566	0.049
Bladder	5	2304/2253	0.94 (0.74–1.20)	0.621	0.112	0.95 (0.75–1.21)	0.686	0.083
Head and neck	3	1429/1954	0.90 (0.67–1.22)	0.510	0.194	0.88 (0.67–1.16)	0.364	0.240
Colorectal	3	743/879	0.97 (0.59–1.58)	0.900	0.372	0.92 (0.57–1.48)	0.720	0.262
NHL	3	2105/1957	1.07 (0.83–1.38)	0.594	0.238	1.03 (0.80–1.32)	0.839	0.345
Others	13	3383/4931	1.02 (0.86–1.21)	0.850	0.384	1.03 (0.89–1.19)	0.696	0.244
**Ethnicity**								
Caucasian	29	13316/15586	0.99 (0.89–1.10)	0.814	0.287	0.98 (0.90–1.08)	0.709	0.452
African-American	4	1318/1330	1.11 (0.89–1.38)	0.365	0.020	1.03 (0.86–1.24)	0.747	0.016
Asian	6	2314/2443	0.90 (0.76–1.06)	0.216	0.292	0.93 (0.82–1.06)	0.262	0.091
Others	10	6542/7809	1.00 (0.88–1.15)	0.982	0.878	0.98 (0.90–1.07)	0.679	0.852
**Source of controls**								
Hospital	27	9787/11586	0.95 (0.85–1.05)	0.298	0.186	0.97 (0.89–1.05)	0.426	0.129
Population	20	10333/11150	1.02 (0.91–1.14)	0.732	0.325	0.99 (0.89–1.09)	0.795	0.427
Others	2	3370/4432	1.02 (0.85–1.23)	0.810	0.853	0.98 (0.90–1.08)	0.706	0.795
**Sample size**								
<500	33	7469/10388	0.98 (0.87–1.11)	0.755	0.055	0.96 (0.86–1.06)	0.396	0.067
500–1000	11	8170/7890	1.02 (0.90–1.15)	0.781	0.704	1.01 (0.91–1.11)	0.923	0.649
>1000	5	7851/8890	0.96 (0.84–1.09)	0.516	0.893	0.97 (0.90–1.05)	0.474	0.916

a *P* value of the Z-test for odds ration test.

b *P* value of the Q-test for heterogeneity test.

c Fixed effects model.

### Heterogeneity and Sensitivity Analyses

There were no between-study heterogeneity among overall studies of the *ERCC5* Asp1104His polymorphism in the recessive genetic models (χ^2^ = 54.45, *P* = 0.242 for heterogeneity test and χ^2^ = 53.86, *P* = 0.260 for heterogeneity test for His/His vs. Asp/Asp and His/His vs. Asp/His + Asp/Asp, respectively). In the sensitivity analyses, the influence of each study on the pooled OR was checked by repeating the meta-analysis while omitting each study, one at a time. This procedure validated the stability of our results. Furthermore, the inclusion of 7 studies, whose genotype distributions among the controls deviated from HWE, affected between-study heterogeneity for His/His vs. Asp/Asp (χ^2^ = 72.21, P = 0.060), but did not influence the result of the meta-analysis significantly: His/His vs. Asp/Asp: OR = 1.00, 95% CI: 0.93–1.09. His/His vs. Asp/His + Asp/Asp: OR = 0.98, 95% CI: 0.93–1.03.

### Publication Bias

We conducted Begg’s funnel plot and Egger’s test to access the publication bias of all included studies. The shape of the funnel plot seemed symmetrical (**Figure S3**), suggesting that there was no obvious publication bias. Furthermore, the Egger’s test further provided statistical evidence that there was no significant publication bias in this meta-analysis (the Egger’s test: His/His vs. Asp/Asp: *P* = 0.897, His/His vs. Asp/His + Asp/Asp: *P* = 0.749).

### Correlation Analysis for *ERCC5* mRNA Expression and Asp1104His Genotypes

**Figure 3:**
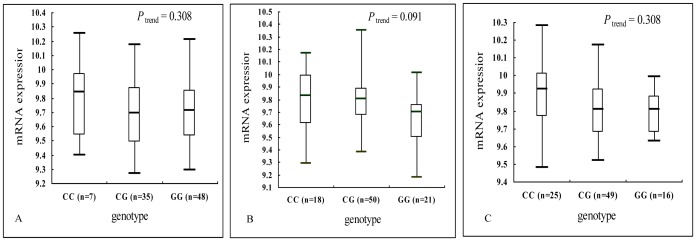
Correlation between Asp1104His and *ERCC5* mRNA expression for different populations. A : CEU, 90 Utah residents from northern and western Europe; **B**: Asians, 45 unrelated Han Chinese in Beijing (CHB) and 45 unrelated Japanese in Tokyo (JPT); **C**: YRI, 90 Yoruba in Ibadan, Nigeria.

In the genotype-phenotype correlation analysis using the lymphoblastoid cell lines derived from peripheral lymphocytes from 270 people, we found no trend for the allele effect on *ERCC5* mRNA expression for Europeans (*P*_ trend_ = 0.308), Asians (*P*_trend_ = 0.091) and Africans (*P*_trend_ = 0.308) (**Figure 3**).

## Discussion

On the basis of eligible 49 case-control studies with a total of 23490 cancer cases and 27168 controls, our meta-analysis comprehensively evaluated the association between the *ERCC5* Asp1104 His polymorphism and risk of different types of cancers, and we did not find statistical evidence for such an association in the recessive genetic models as shown in the XP syndrome. Similarly, in subgroup analyses, we consistently showed no statistical association between the polymorphism and cancer risk in any of the subgroups. Furthermore, the observed null associations were supported by the data that the variant genotypes of the SNP were not associated with mRNA expression levels of *ERCC5* in the lymphoblastoid cell lines.

DNA repair deregulation is a crucial factor in the multistep process of carcinogenesis, and the *ERCC5* gene is a vital component of the DNA repair machinery. It has been observed that deficiency of *ERCC5* may result in severe autosomal recessive diseases including XP, CS and TTD [14] characterized by solar hypersensitivity of the skin, high predisposition for developing cancers (mainly epithelial and melanoma) on areas exposed to sunlight. Furthermore, studies have suggested that *ERCC5* SNPs are associated with development of some cancers, such as breast cancer [44] and smoking-related cancers [23], [24]. These suggest a possible link between the *ERCC5* function and development of cancer. The biological mechanisms of the *ERCC5* gene in carcinogenesis may be complicated, among which nsSNPs, leading to an amino acid change in the protein product and modulating the individual DNA repair capacity phenotype [113], [114], may account for some of the known genetic variations related to risk of cancers. However, our meta-analysis suggests that there is no statistical evidence for an association between the *ERCC5* Asp1104His polymorphism and overall cancer risk, which is consistent with the previous two meta-analyses conducted in breast cancer and melanoma, respectively. The former included some studies departure from HWE in the control population, and the latter only contained three studies. Although we excluded the inappropriate studies and expanded the sample size, the null results were not altered. Furthermore, as far as our knowledge is concerned, none of the SNPs in NER have ever been identified as susceptibility locus in the published genome-wide association studies (GWAS) for common diseases including cancers based on common SNPs, which are similar to our results. This is a challenge to the theory of common variants and common diseases [115]. It is likely that, as NER genes are considered susceptibility genes, the role of NER variants in cancer development may be dependent on the degree of exposures that cause damage to DNA. Therefore, without detailed information about such exposures for further adjustment or stratification, the results of the observed associations may be either biased or masked. For example, XP patients can drastically reduce risk of developing skin cancer by avoiding exposure to sunlight. Another possibility is that the common variants are unlikely to have a significant biological effect. For common variants, in most cases, the disease-associated variant itself is unlikely to be functionally relevant [115]. The third possibility is that the genetic risk of cancer conferred by the common variants is very modest and the penetrance of the variants is very small, which means that even if the polymorphism is crucial for carcinogenesis, extremely large-scale evidence would be necessary to establish with high confidence the presence of specific associations. The inclusion of rare variants and larger samples in future genome-wide association studies would help to reveal low-penetrance susceptibility loci that are more likely to be associated with cancer risk.

Furthermore, we did not observe biological evidence for the effect of this SNP on the gene expression in terms of mRNA levels, which biological support for the result of no association. Although a sequence homology-based tool predicted this *ERCC5* polymorphism to be a deleterious substitution [116], and computational algorithms by SIFT and SNPs3D tools aslo identified the polymorphism with some functional implications (http://compbio.cs.queensu.ca/F-SNP/), such a potentially functional relevance have not been validated experimentally to date. Diversities of variant-related risk associations in various kinds of cancer may result from different mechanisms of carcinogenesis among different cancer types. Although some studies have discovered some sequence variants in the region of chromosome 5p15.33 and 8q24 that are associated with risk of different cancer types [117]–[122], it is still uncertain whether the same polymorphism may have non-specific effect on different types of cancer. Therefore, further functional studies should be undertaken to explore the mechanism underlying the variant-related associations with cancer risk.

It would be hard to interpret results, if significant heterogeneity were present. However, in this meta-analysis, we did not find any obvious heterogeneity and publish bias across studies. Nevertheless, some limitations should be addressed. Firstly, although funnel plot and Egger’s test show no publication bias, selection bias could have occurred because only studies published in English and Chinese were included. Secondly, because the reference groups were not uniformly defined, some selected population-based controls and some used hospital-based cancer-free controls, non-differential misclassification bias is possible; in addition, some control groups may not be representative of the general population. Thirdly, our results were based on unadjusted OR estimates, because not all published studies presented adjusted ORs or when they did, the ORs were not adjusted by the same potential confounders, such as age, sex and exposure variables. Thus, more comprehensive individual datasets are needed to allow for the adjustment by some co-variants and further evaluation of potential gene-environmental interactions for susceptibility to cancer. Fourthly, although the sample size of our study was relatively large, the statistical power was still limited in the analyses of subgroups with small sample sizes, particularly when the effect size is small. Therefore, studies with larger sample sizes with sufficient large subgroups should be undertaken to validate our findings.

In summary, our meta-analysis shows that the *ERCC5* Asp1104His polymorphism appeared to be unlikely to confer susceptibility to cancers. Further well-designed studies with larger sample sizes will be necessary to validate the findings in the present meta-analysis.

## Supporting Information

Figure S1 ***ERCC5*** **gene map labeled with nine SNPs have been studied for associations with cancer risk.** (A) Six SNPs are located in the coding region, among which four are nsSNPs, of which two are synonymous SNPs; two SNPs are located in the 5′ untranslated region, and one SNP is located in the 3′ untranslated region; six SNPs are tagging SNPs. (B) Nine SNPs with a minor allelic frequency in different populations obtained from the dbSNP database.
(TIF)

Figure S2 **Flow chart of included studies for this meta-analysis.**
(TIF)

Figure S3 **Funnel plot analysis to detect publication bias for** ***ERCC5*** **Asp1104His under the recessive genetic models (A, His/His vs. Asp/Asp and B, His/His vs. Asp/His + Asp/Asp) for all 44 studies.** Each point represents an individual study for the indicated association.
(TIF)

Table S1 **Summary of 24 SNPs of the** ***ERCC5/XPG*** **gene that have been studied for their associations with cancer risk.**
(DOCX)

Table S2 **Summary of Studied SNPs in the eight NER genes reviewed in all published meta-analysis.**
(DOCX)

Table S3 **Characteristics of the 44 references included in the meta-analysis for ERCC5 Asp1104His.**
(DOCX)
