# Supplementary figures and images for: Association between the ERCC5 Asp1104His Polymorphism and Cancer Risk: A Meta-Analysis
Source: PLoS One. 2012 Jul 18;7(7):e36293. doi: 10.1371/journal.pone.0036293 (PMC3399856; doi:10.1371/journal.pone.0036293)

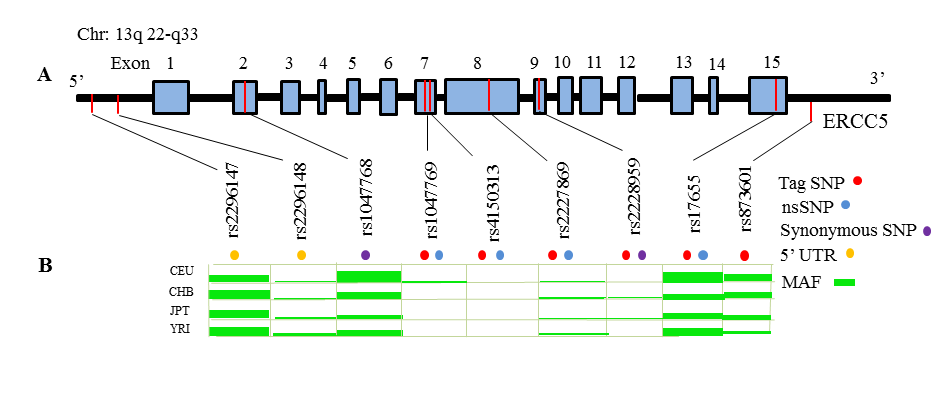

Supplement: Figure S1 — ERCC5 gene map labeled with nine SNPs have been studied for associations with cancer risk. (A) Six SNPs are located in the coding region, among which four are nsSNPs, of which two are synonymous SNPs; two SNPs are located in the 5′ untranslated region, and one SNP is located in the 3′ untranslated region; six SNPs are tagging SNPs. (B) Nine SNPs with a minor allelic frequency in different populations obtained from the dbSNP database. (TIF) [file pone.0036293.s001.tif]

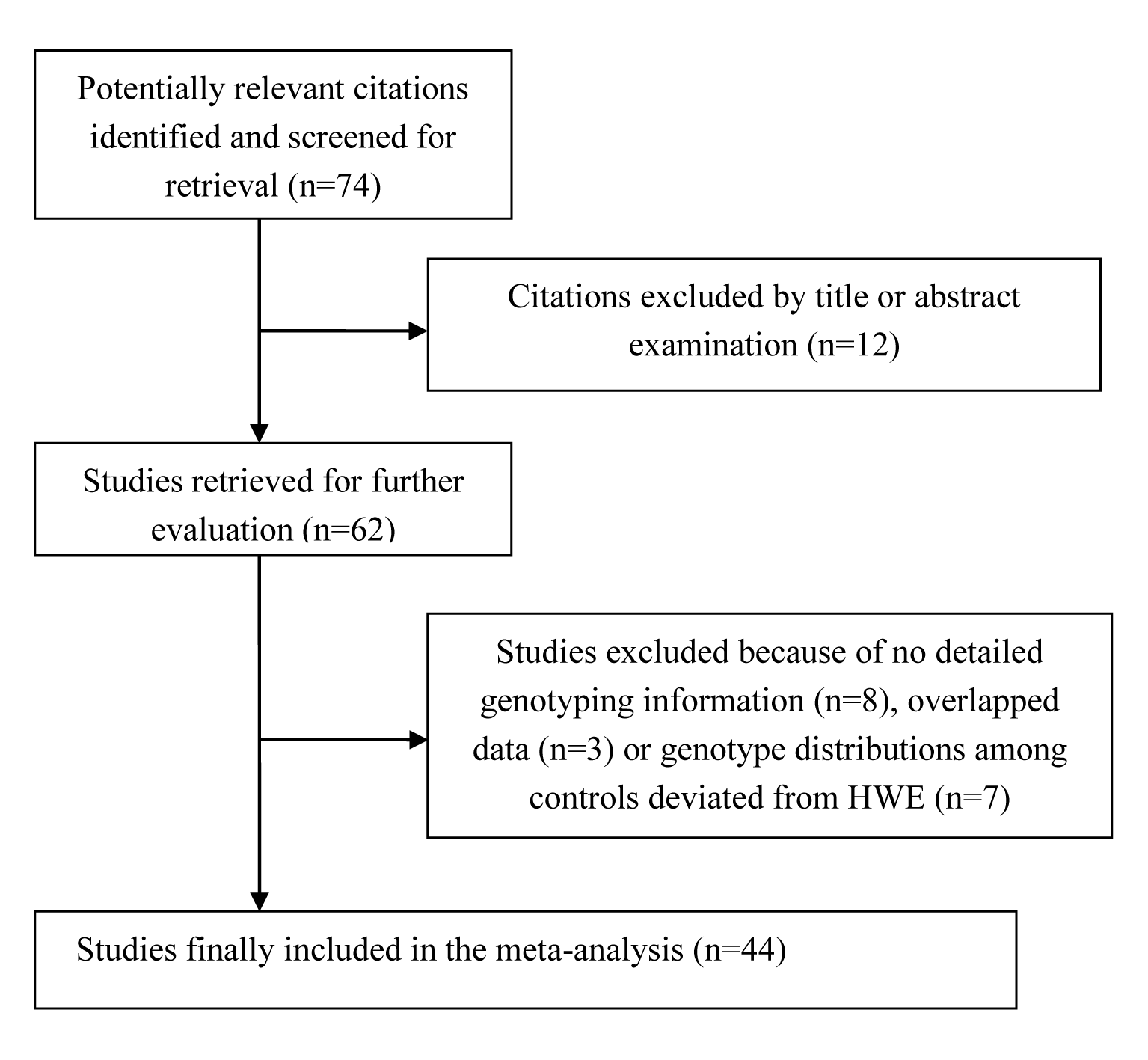

Supplement: Figure S2 — Flow chart of included studies for this meta-analysis. (TIF) [file pone.0036293.s002.tif]

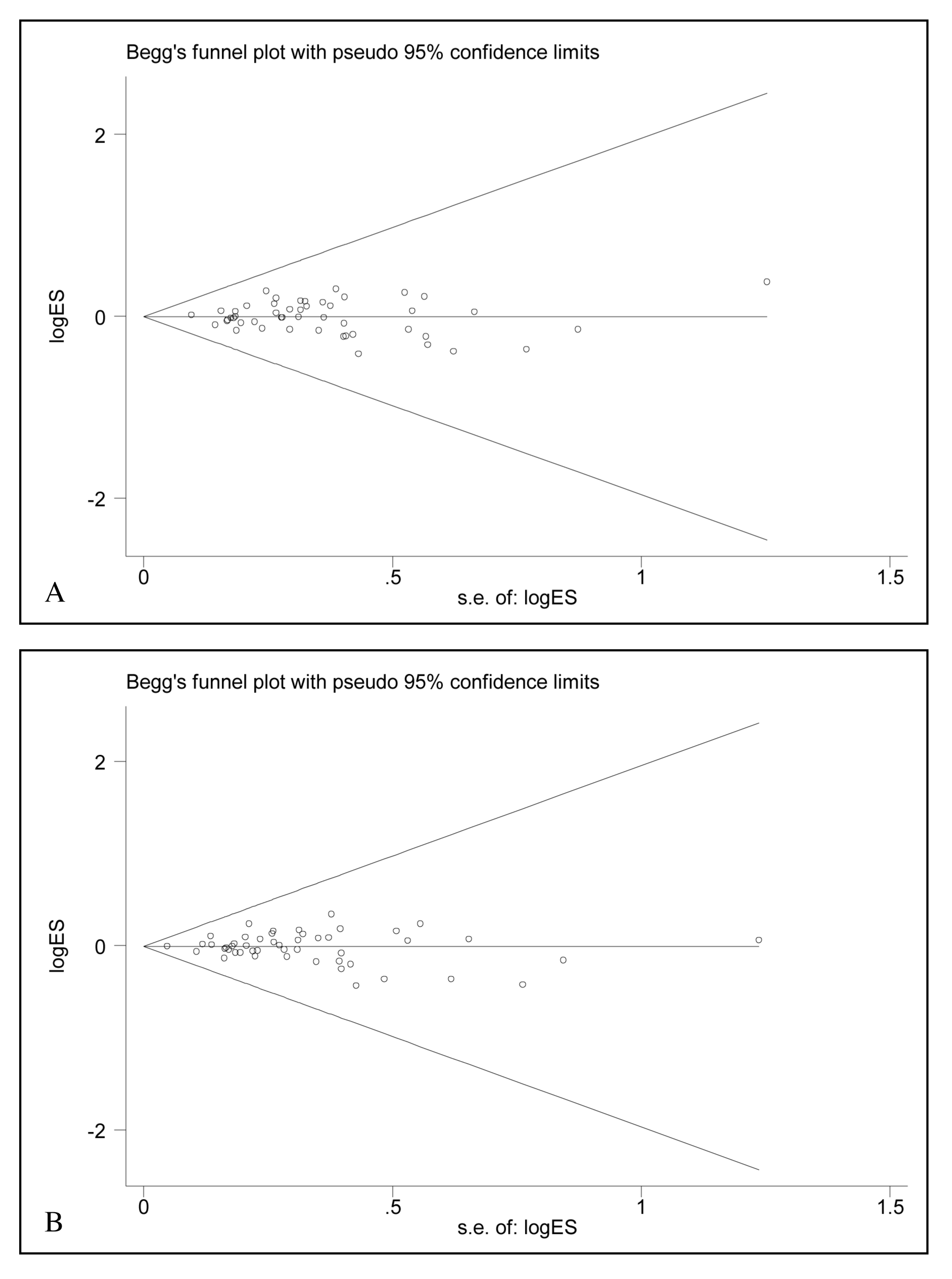

Supplement: Figure S3 — Funnel plot analysis to detect publication bias for ERCC5 Asp1104His under the recessive genetic models (A, His/His vs. Asp/Asp and B, His/His vs. Asp/His + Asp/Asp) for all 44 studies. Each point represents an individual study for the indicated association. (TIF) [file pone.0036293.s003.tif]
