# Supplementary material for: Association between the ERCC5 Asp1104His Polymorphism and Cancer Risk: A Meta-Analysis
Source: PLoS One. 2012 Jul 18;7(7):e36293. doi: 10.1371/journal.pone.0036293 (PMC3399856; doi:10.1371/journal.pone.0036293)
Supplement: Table S1 — Summary of 24 SNPs of the ERCC5/XPG gene that have been studied for their associations with cancer risk. (DOCX) [file pone.0036293.s004.docx]

**Table S1**. Summary of 24 SNPs of the *ERCC5/XPG* gene that have been studied for their associations with cancer risk

| Author | Country | Ethnicity | Cancer type | Sample sizes (Cases/controls) | Source of controls | Genotype method | Main findings | Polymorphisms |
| --- | --- | --- | --- | --- | --- | --- | --- | --- |
| Shen, 2005^[89]^ | China | Asian | Lung cancer | 118/112 | PB | TaqMan | No significant effect on cancer risk | rs1047768, rs2227869, rs2228959 |
| Garcia-Closas, 2006 ^[85]^ | Spanish | Caucasian | Bladder cancer | 1103/1094 | HB | Sequencing | No significant effect on cancer risk | rs1047768, rs1047769 |
| Michiels, 2007^[56]^ | France | Caucasian | Lung cancer | 147/172 | HB | Mixed | No significant effect on cancer risk | rs4771436, rs732321, rs2018836, rs3759500, rs3818356 |
| Hooker, 2008^[106]^ | USA | African-  Amercian | Prostate cancer | 254/304 | HB | MassARRAY | **rs2296148 TT carriers 0.12 (0.03–0.48)**, no significant effect on cancer risk for other SNPs | rs2227869, rs2296148, rs4150313 |
| Rajaraman, 2008^[75]^ | USA | Caucasian | Breast cancer | 852/1082 | PB | TaqMan | No significant effect on cancer risk | rs1047769 |
| Abbasi, 2009^[94]^ | Germany | Caucasian | Laryngeal cancer | 248/647 | PB | TaqMan | No significant effect on cancer risk | rs1047768 |
| Hussain, 2009^[102]^ | China | Asian | Gastric cancer | 343/213 | PB | SNPlex™ | **rs1047768 TC carriers 0.65 (0.41–1.03), rs2227869 GC carriers 0.30 (0.13–0.67)** | rs1047768, rs2227869 |
| Doherty, 2011^[105]^ | USA | Mixed | Endometrial  cancer | 722/726 | PB | SNaPshot  /SNPlex | **rs4150386 C allele 0.68 (0.53-0.87)**, no significant effect on cancer risk for other SNPs | rs4150386, rs2296147, rs4150351, rs4150355, rs4150383, rs4150393, rs3818356, rs4150261, rs4150276, rs4150375 |
| Hongxia,2011^[95]^ | USA | Caucasian | SCCHN | 1059/1066 | PB | SNPlex  /PCR-RFLP | No significant effect on cancer risk | rs1047768, rs2227869, rs2296147, rs4150351, rs4150355, rs4150383, rs4150386, rs4150393, rs4771436, rs873601, rs2094258 |

*HB,* Hospital based; *PB*, Population based; *PCR,* polymerase chain reaction; *RFLP,* Restriction fragment length polymorphisms polymerase chain reaction; *SCCHN,* Squamous cell carcinoma of the head and neck; Some of the studies were marked as ‘mixed’ ethnic, because the genotyping data was mixed from different populations.
