# Supplementary material for: Association between the ERCC5 Asp1104His Polymorphism and Cancer Risk: A Meta-Analysis
Source: PLoS One. 2012 Jul 18;7(7):e36293. doi: 10.1371/journal.pone.0036293 (PMC3399856; doi:10.1371/journal.pone.0036293)
Supplement: Table S2 — Summary of Studied SNPs in the eight NER genes reviewed in all published meta-analysis. (DOCX) [file pone.0036293.s005.docx]

**Table S2.** Summary of Studied SNPs in the eight NER genes reviewed in all published meta-analysis.

| Gene | Polymorphism | Function | Cancer type | Main findings, ORs (95% CI) | Reference^f^ |
| --- | --- | --- | --- | --- | --- |
| *ERCC1* |  |  |  |  |  |
|  | Asn118Asn (rs11615) | synonymous | Mixed | No significant effect on cancer risk | Li,2007 ^[29]^ |
|  | Asn118Asn (rs11615) | synonymous | Lung cancer | No significant effect on cancer risk | Cao,2011 ^[30]^ |
|  | C8092A(rs3212986) | 3'UTR | Mixed | No significant effect on cancer risk | Li,2007 ^[29]^ |
|  | C8092A(rs3212986) | 3'UTR | Lung cancer | No significant effect on cancer risk | Cao,2011 ^[30]^ |
| *XPA* |  |  |  |  |  |
|  | G23A (rs1800975) | 5'UTR | Lung cancer | 1.36 (1.13-1.64)^b^ for lung cancer | Vineis,2009 ^[31]^ |
|  | G23A (rs1800975) | 5'UTR | Lung cancer | 1.28 (1.12-1.47)^a^ for all population, 1.33 (1.06-1.67)^b^ and1.27 (1.01-1.59)^d^ for Asians | Qian,2011 ^[33]^ |
| *XPC* |  |  |  |  |  |
|  | Ala499Val (rs2228000) | non-synonymous | Mixed | 1.24 (1.08-1.42)^a^ for overall cancer , 1.27 (1.11-1.45)^b^ for bladder cancer | Qiu,2008 ^[34]^ |
|  | Ala499Val (rs2228000) | non-synonymous | Bladder cancer | 1.10 (1.00-1.21)^e^ for bladder cancer | Stern,2009 ^[35]^ |
|  | Ala499Val (rs2228000) | non-synonymous | Breast cancer | No significant effect on cancer risk | Zheng,2011 ^[36]^ |
|  | Lys939Gln (rs2228001) | non-synonymous | Mixed | 1.16 (1.05-1.28)^a^ for overall cancer , 1.17 (1.06-1.28)^b^ for lung cancer | Qiu,2008 ^[34]^ |
|  | Lys939Gln (rs2228001) | non-synonymous | Bladder cancer | No significant effect on cancer risk | Stern,2009 ^[35]^ |
|  | Lys939Gln (rs2228001) | non-synonymous | Breast cancer | No significant effect on cancer risk | Zheng,2011 ^[36]^ |
|  | PAT+/- | Intron | Mixed | 0.86 (0.76-0.98)^c^ and 0.87 (0.77-0.99)^d^ for Asians | Qiu,2008 ^[34]^ |
|  | PAT+/- | Intron | Breast cancer | 1.41 (1.05-1.89)^b^ for breast cancer | Zheng,2011 ^[36]^ |
| *ERCC2/XPD* | |  |  |  |  |
|  | Lys751Gln (rs13181) | non-synonymous | Mixed | 1.61(1.16-2.25)^a^ and 1.34 (1.10-1.64)^c^ for esophageal cancer, 1.83(1.21-2.75)^a^ for acute lymphoblastic leukemia | Wang,2008 ^[37]^ |
|  | Lys751Gln (rs13181) | non-synonymous | Cutaneous melanoma | 1.12 (1.03-1.21)^e^ for cutaneous melanoma | Mocellin,2009 ^[39]^ |
|  | Lys751Gln (rs13181) | non-synonymous | Lung cancer | 1.27(1.10-1.46)^a^ for all population, 1.24 (1.06-1.45)^a^ for Caucasians | Kiyohara,2010 ^[32]^ |
|  | Lys751Gln (rs13181) | non-synonymous | Bladder cancer | 1.12 (1.01-1.26)^b^ for bladder cancer | Li,2009 ^[38]^ |
|  | Lys751Gln (rs13181) | non-synonymous | Breast cancer | No significant effect on cancer risk | Pabalan,2010 ^[40]^ |
|  | Lys751Gln (rs13181) | non-synonymous | Breast cancer | Elevated cancer risk in all models, 1.14 (1.02-1.27)^b^ | Qiu,2010 ^[41]^ |
|  | Lys751Gln (rs13181) | non-synonymous | Gastric cancer | 2.43 (1.70-3.48)^b^ among Asians | Chen,2011 ^[42]^ |
|  | Lys751Gln (rs13181) | non-synonymous | Esophageal cancer | 1.25 (1.01-1.55)^a^ for esophageal cancer | Yuan,2011 ^[43]^ |
|  | Asp312Asn (rs1799793) | non-synonymous | Mixed | 1.24 (1.06-1.46)^c^ for overall cancer | Wang,2008 ^[37]^ |
|  | Asp312Asn (rs1799793) | non-synonymous | Bladder cancer | Increased risk for bladder cancer under all genetic models. | Li,2009 ^[38]^ |
|  | Asp312Asn (rs1799793) | non-synonymous | Lung cancer | 1.19 (1.03-1.38)^a^ for lung cancer | Kiyohara,2010 ^[32]^ |
|  | Asp312Asn (rs1799793) | non-synonymous | Breast cancer | No significant effect on cancer risk | Pabalan,2010 ^[40]^ |
|  | Asp312Asn (rs1799793) | non-synonymous | Gastric cancer | 1.36 (1.04-1.77)^b^ among Asians | Chen,2011 ^[42]^ |
|  | Arg156Arg (rs238406) | synonymous | Cutaneous melanoma | No association with melanoma risk | Mocellin,2009 ^[39]^ |
| *ERCC4/XPF* | |  |  |  |  |
|  | Ser835Ser (rs1799801) | synonymous | Bladder cancer | No significant effect on cancer risk | Yuan,2011 ^[43]^ |
|  | Arg415Gln (rs1800067) | non-synonymous | Breast cancer | 5.20 (2.08-12.95)^a^ for Caucasians | Ding,2011 ^[44]^ |
| *ERCC5/XPG* | |  |  |  |  |
|  | Asp1104His (rs17655) | non-synonymous | Cutaneous melanoma | No significant effect on cancer risk | Mocellin,200^[39]^ |
|  | Asp1104His (rs17655) | non-synonymous | Breast cancer | No significant effect on cancer risk | Ding,2011 ^[44]^ |

**^a^** Odds ratios (ORs) and 95% confidence interval (CI) for homozygote model for *XPA* G23A GG vs. AA or AA vs. GG; *XPC* Ala499Val Val/Val vs. Ala/Ala, Lys939Gln Gln/Gln vs. Lys/Lys; *ERCC2/XPD* Asp312Asn Asn/Asn vs. Asp/Asp; *ERCC2/XPD* Lys751 Gln Gln/Gln vs. Lys/Lys; *ERCC4/XPF* Arg415Gln Gln/Gln vs. Arg/Arg.

**^b^** ORs and 95%CI for recessive genetic model for *XPA* G23A AA vs. (GG+GA); *XPC* Ala499Val Val/Val vs. (Ala/Val + Ala/Ala), Lys939Gln Lys/Lys+Lys/Gln vs. Gln/Gln; PAT+/- (+/–) + (–/–) versus (+/+); *ERCC2/XPD* Asp312Asn Asn/Asn vs. (Asn/Asp+Asp/Asp); *ERCC2/XPD* Lys751 Gln/Gln vs. (Gln/Lys + Lys/Lys).

**^c^** ORs and 95%CI for heterozygote model for XPC PAT+/- (+/–) vs. (–/–); *ERCC2/XPD* Asp312Asn Asp/Asn vs. Asp/Asp; *ERCC2/XPD* Lys751 Lys/Gln vs. Lys/Lys.

**^d^** ORs and 95%CI for dominant genetic model for XPA G23A (AA+GA) vs. GG; *XPC* PAT+/- (+/–) + (+/+) vs. (–/–).

**^e^** ORs and 95%CI for variant allele.

^f^ We selected the most recent study that included the largest number of individuals in the published meta-analysis, if studies had overlapping subjects.

UTR, untranslated region; NSCLC, non-small cell lung cancer; Mixed, different cancer types
