# Supplementary material for: Association between the ERCC5 Asp1104His Polymorphism and Cancer Risk: A Meta-Analysis
Source: PLoS One. 2012 Jul 18;7(7):e36293. doi: 10.1371/journal.pone.0036293 (PMC3399856; doi:10.1371/journal.pone.0036293)
Supplement: Table S3 — Characteristics of the 44 references included in the meta-analysis for ERCC5 Asp1104His. (DOCX) [file pone.0036293.s006.docx]

**Table S3.** Characteristics of the 44 references included in the meta-analysis for *ERCC5* Asp1104His

| Author | Country | Ethnicity | Cancer type | Sample sizes (Cases/controls) | Source of controls | Genotype method | MAF (controls) | HWE (controls) | Main findings |
| --- | --- | --- | --- | --- | --- | --- | --- | --- | --- |
| Kumar, 2003^[69]^ | Finland | Caucasian | Breast cancer | 220/308 | HB | PCR-RFLP | 0.24 | 0.54 | 1104His significantly increased breast cancer risk |
| Mechanic, 2006^[70]^ | USA | African-American, Caucasian | Breast cancer | 757/674  1249/1133 | PB | TaqMan | 0.42  0.23 | 0.50  0.69 | No association with cancer risk |
| Shen, 2006^[71]^ | USA | Caucasian | Breast cancer | 154/151 | FB | TaqMan | 0.25 | 0.27 | No association with cancer risk |
| Crew, 2007^[72]^ | USA | Caucasian | Breast cancer | 999/1051 | PB | Mass spectrometry | 0.26 | 0.85 | No association with cancer risk |
| Jorgensen, 2007^[73]^ | USA | Caucasian | Breast cancer | 264/275 | PB | TaqMan | 0.23 | 0.78 | No association with cancer risk |
| Smith, 2008^[74]^ | USA | African-American | Breast cancer | 52/75 | HB | Mass Array System | 0.51 | 0.91 | No association with cancer risk |
| Rajaraman, 2008^[75]^ | USA | Caucasian | Breast cancer | 819/1079 | PB | TaqMan | 0.21 | 0.42 | 1104His marginally increased breast cancer risk |
| Hsu, 2010^[76]^ | Taiwan | Asian | Breast cancer | 401/531 | HB | TaqMan | 0.47 | 0.06 | 1104Asp variant increased breast cancer risk |
| Blankenburg, 2005^[77]^ | Germany | Caucasian | Cutaneous melanoma | 293/374 | PB | PCR-RFLP | 0.21 | 0.78 | No association with cancer risk |
| Li, 2006^[78]^ | USA | Caucasian | Cutaneous melanoma | 602/603 | HB | PCR-RFLP | 0.22 | 0.80 | No association with cancer risk |
| Povey, 2007 ^[79]^ | UK | Caucasian | Cutaneous melanoma | 507/441 | PB | PCR-RFLP | 0.24 | 0.89 | No association with cancer risk |
| Figl, 2010^[80]^ | German, Spain | Caucasian | Cutaneous melanoma | 1186/1274 | HB | TaqMan | 0.25 | 0.43 | No association with cancer risk |
| Ibarrola-Villava, 2011^[81]^ | Spain | Caucasian | Malignant melanoma | 602/380 | HB | Mass Spectrometer | 0.25 | 0.84 | No association with cancer risk |
| Thirumaran, 2006^[82]^ | Mixed | Caucasian | Skin basal cell carcinoma | 529/533 | HB | TaqMan | 0.22 | 0.25 | No association with cancer risk |
| Wang, 2007^[83]^ | USA | Caucasian | Skin basal cell carcinoma | 86/329, 104/329 | HB | PCR-RFLP | 0.21 | 0.12 | / |
| Sanyal, 2004^[84]^ | Stockholm | Caucasian | Bladder cancer | 299/284 | HB | PCR-RFLP | 0.23 | 0.10 | Asp/Asp decreased risk of bladder cancer |
| Garcia-Closas, 2006^[85]^ | Spain | Caucasian | Bladder cancer | 1141/1136 | HB | Sequencing | 0.27 | 0.84 | No association with cancer risk |
| Wu,2006^[86]^ | USA | Caucasian | Bladder cancer | 615/600 | HB | Taqman | 0.21 | 0.06 | No association with cancer risk |
| Narter, 2009^[87]^ | Turkey | Turk | Bladder cancer | 56/40 | HB | PCR--RFLP | 0.31 | 0.50 | No association with cancer risk |
| Rouissi1, 2011^[88]^ | Tunisia | Tunisian | Bladder cancer | 193/193 | HB | PCR--RFLP | 0.33 | 0.85 | No association with cancer risk |
| Shen, 2005^[89]^ | China | Asian | Lung cancer | 116/109 | PB | TaqMan | 0.56 | 0.13 | No association with cancer risk |
| Sakiyama, 2005^[90]^ | Japan | Asian | Lung cancer | 1002/685 | HB | Pyrosequencing | 0.58 | 0.90 | 1104His decreased lung cancer risk, but the association was eliminated by adjustment for other risk factors |
| Hung, 2008^[91]^ | Multiple | Mixed | Lung cancer | 3216/4281 | Mixed | Unknown | 0.76 | 0.01 | No association with cancer risk |
| Chang, 2008^[92]^ | USA | Latinos, African-Americans | Lung cancer | 113/299, 255/280 | PB | Illumina | 0.33, 0.42 | 0.56, 0.86 | His/His elevated risk of lung cancer among African Americans. No association among Latinos |
| Sugimura, 2006^[93]^ | Japan | Asian | OSCC | 122/241 | HB | PCR-RFLP | 0.55 | 0.35 | No association with cancer risk |
| Abbasi, 2009^[94]^ | Germany | Caucasian | Laryngeal cancer | 248/647 | PB | TaqMan | 0.23 | 0.78 | GC carriers exhibited a significantly increased risk of laryngeal cancer |
| Hongxia,2011^[95]^ | USA | Caucasian | SCCHN | 1059/1066 | PB | SNPlex/PCR-RFLP | 0.22 | 0.10 | No association with SCCHN risk |
| Pardini, 2008^[96]^ | Czech Republic | Caucasian | Colorectal cancer | 532/532 | HB | PCR-RFLP | 0.19 | 0.21 | Asp/His variant was associated with a borderline increased risk of colorectal cancer |
| Gil, 2011^[97]^ | Poland | Caucasian | Colorectal cancer | 132/100 | HB | PCR-RFLP | 0.21 | 0.62 | No association with cancer risk |
| Canbay, 2011^98]^ | Turkey | Caucasian | Colorectal cancer | 79/247 | HB | PCR-RFLP | 0.23 | 0.35 | No association with cancer risk |
| Shen, 2006^[99]^ | USA | Mixed | NHL | 464/550 | PB | TaqMan | 0.21 | 0.15 | 1104His increased risk of NHL |
| Hill,2006^[100]^ | USA | Mixed | NHL | 1101/923 | PB | Unknown | 0.26 | 0.07 | No association with cancer risk |
| Shen,2007^[101]^ | Australia | Mixed | NHL | 540/484 | PB | Taqman | 0.22 | 0.48 | No association with cancer risk |
| Hussain, 2009^[102]^ | China | Asian | Gastric cancer | 173/370 | PB | SNPlex™ | 0.50 | 1.00 | No association with cancer risk |
| Canbay, 2010^[103]^ | Turkey | Turk | Gastric cancer | 40/247 | HB | PCR-RFLP | 0.23 | 0.35 | No association with cancer risk |
| Weiss, 2005^[104]^ | USA | Mixed | Endometrial cancer | 371/420 | PB | SNaPshot | 0.23 | 0.99 | No association with cancer risk |
| Doherty, 2011^[105]^ | USA | Mixed | Endometrial cancer | 714/703 | PB | SNaPshot/SNPlex | 0.24 | 0.27 | No association with cancer risk |
| Hooker, 2008^[106]^ | USA | African-Amercian | Prostate cancer | 254/301 | HB | MassARRAY | 0.56 | 0.48 | No association with cancer risk |
| Morvan, 2006^[107]^ | France | Caucasian | Sarcoma | 93/53 | PB | PCR-RFLP | 0.22 | 0.23 | / |
| Biason, 2011^[108]^ | Italy | Caucasian | Osteosarcoma | 130/250 | HB | Pyrosequencing | 0.25 | 0.90 | No association with cancer risk |
| El-Zein, 2009^[109]^ | USA | Mixed | Hodgkin | 198/219 | PB | Taqman | 0.24 | 0.90 | No association with cancer risk |
| Rajaraman, 2010^[110]^ | USA | Caucasian | Glioma, Meningioma, Acoustic neuroma | 342/468, 121/468, 65/468 | HB | TaqMan | 0.22 | 0.44 | No association with cancer risk |
| Pan, 2009^[111]^ | USA | Caucasian | Esophagus cancer | 382/457 | HB | Taqman | 0.20 | 0.28 | No association with cancer risk |
| Li, 2010^[112]^ | China | Asian | Hepatocellular carcinoma | 500/507 | HB | Taqman | 0.56 | 0.18 | No association with cancer risk |

*HB*, Hospital based; *PB*, Population based; *FB*, Family based; *PCR*, polymerase chain reaction; *RFLP*, Restriction fragment length polymorphisms polymerase chain reaction; *OSCC*, Oral squamous cell carcinoma; *SCCHN*, Squamous cell carcinoma of the head and neck; *NHL*, Non-Hodgkin lymphoma; *HWE*, Hardy-Weinberg equilibrium; *MAF*, minor allelic frequency.
